# Supplementary material for: Adapting Clinical Chemistry Plasma as a Source for Liquid Biopsies
Source: medRxiv. 2026 Feb 26:2025.08.13.25333564. Preprint. [Version 2] doi: 10.1101/2025.08.13.25333564 (PMC12970379; doi:10.1101/2025.08.13.25333564)
Supplement: Supplementary file 1 [file NIHPP2025.08.13.25333564V2-supplement-1.pdf]

medRxiv preprint doi: https://doi.org/10.1101/2025.08.13.25333564; this version posted February 26, 2026. The copyright holder for this preprint (which was not certified by peer review) is the author/funder, who has granted medRxiv a license to display the preprint in perpetuity.
It is made available under a CC-BY 4.0 International license .

SUPPLEMENTARY FIGURES

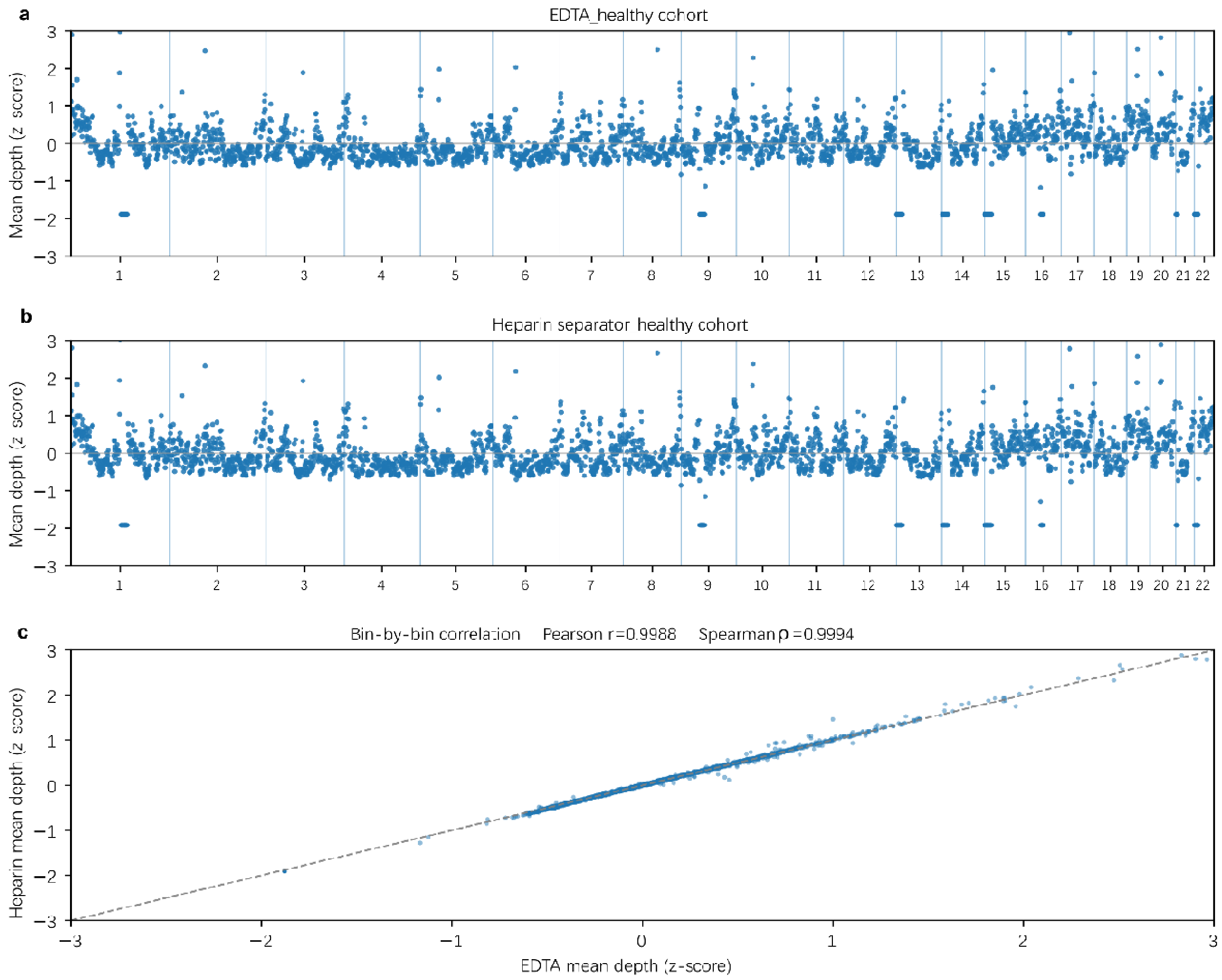

Supplementary Figure 1. Genome-wide coverage comparison between tube types

a. Genome-wide mean sequencing depth across autosomes (Chr1–22) for pooled EDTA plasma samples (n=5). Mean depth was calculated in 1 Mb bins and normalized to z-scores.

b. Genome-wide mean sequencing depth across autosomes (Chr1–22) for pooled Heparin separator plasma samples (n=5).

c. Bin-by-bin comparison of z-scored mean depth between pooled EDTA and pooled heparin separator plasma across all 1 Mb bins on Chr1–22. Each point represents one genomic bin. The dashed line indicates the identity line (y = x).

medRxiv preprint doi: https://doi.org/10.1101/2025.08.13.25333564; this version posted February 26, 2026. The copyright holder for this preprint (which was not certified by peer review) is the author/funder, who has granted medRxiv a license to display the preprint in perpetuity.
It is made available under a CC-BY 4.0 International license .

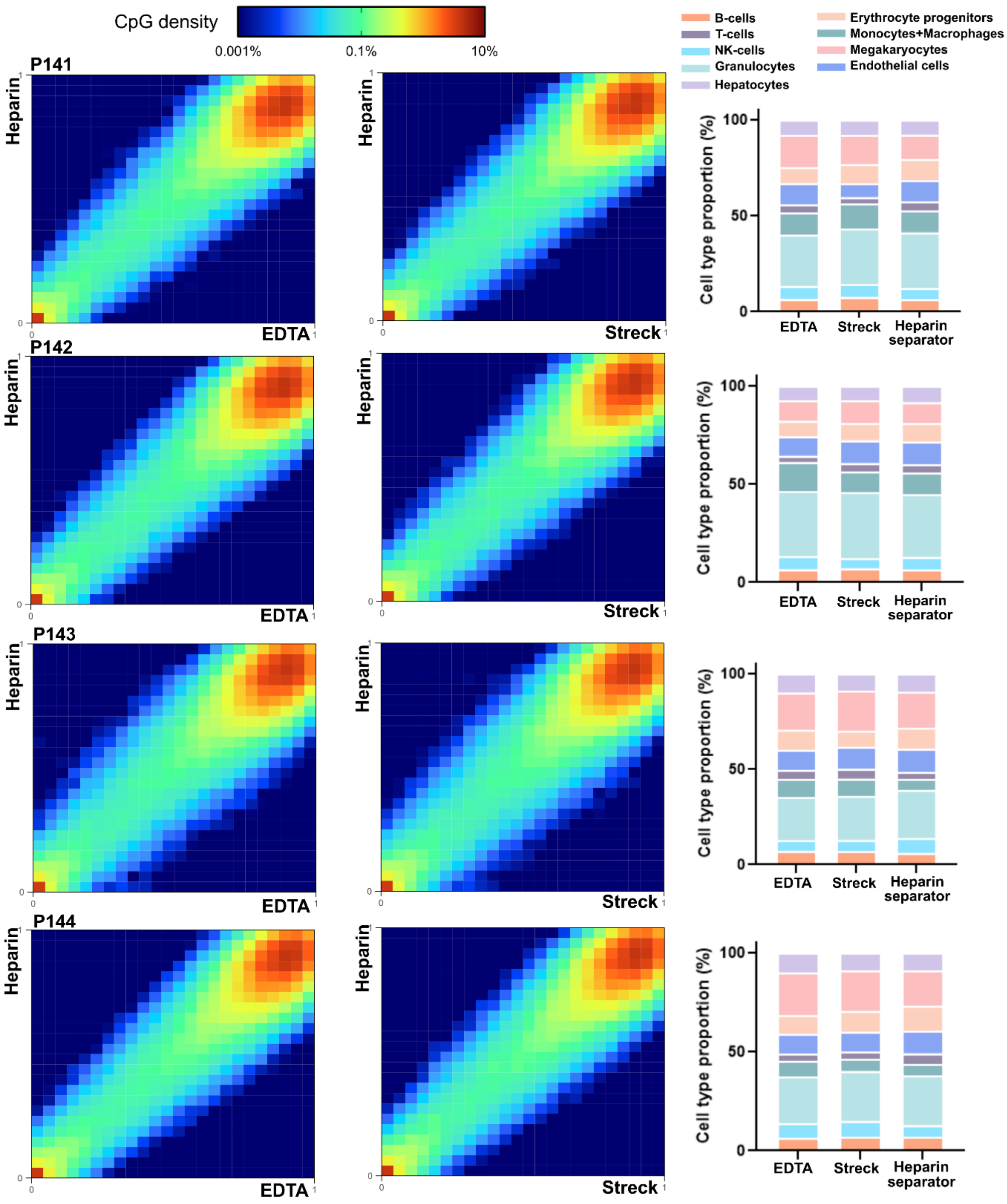

Supplementary Figure 2. Healthy Cohort methylation correlation and cell type deconvolution

Heatmap and correlation of methylation beta values with cell type deconvolution results in paired samples from five healthy donors.

medRxiv preprint doi: https://doi.org/10.1101/2025.08.13.25333564; this version posted February 26, 2026. The copyright holder for this preprint (which was not certified by peer review) is the author/funder, who has granted medRxiv a license to display the preprint in perpetuity.
It is made available under a CC-BY 4.0 International license .

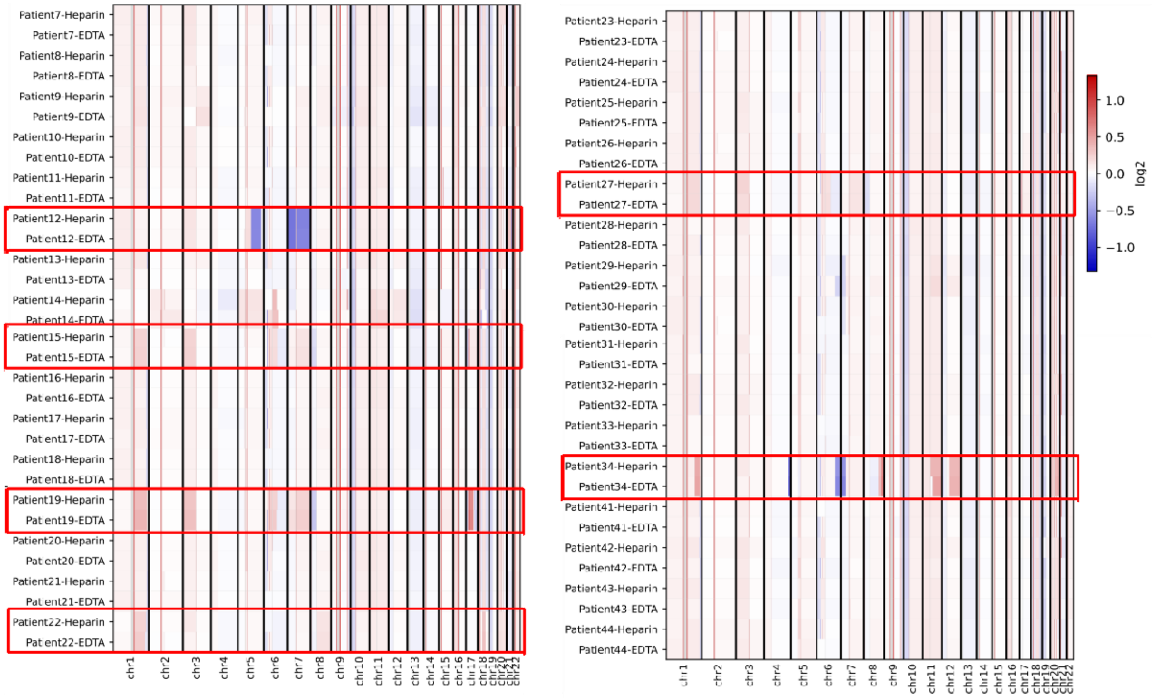

Supplementary Figure 3. CNVkit heatmap across tube types

Genome-wide copy number profiles reanalyzed using CNVkit as a secondary CNV caller. Heatmaps show log2 copy ratio across autosomes for matched EDTA and heparin separator plasma samples. Red boxes highlight CNV positive cases.

medRxiv preprint doi: https://doi.org/10.1101/2025.08.13.25333564; this version posted February 26, 2026. The copyright holder for this preprint (which was not certified by peer review) is the author/funder, who has granted medRxiv a license to display the preprint in perpetuity.
It is made available under a CC-BY 4.0 International license .

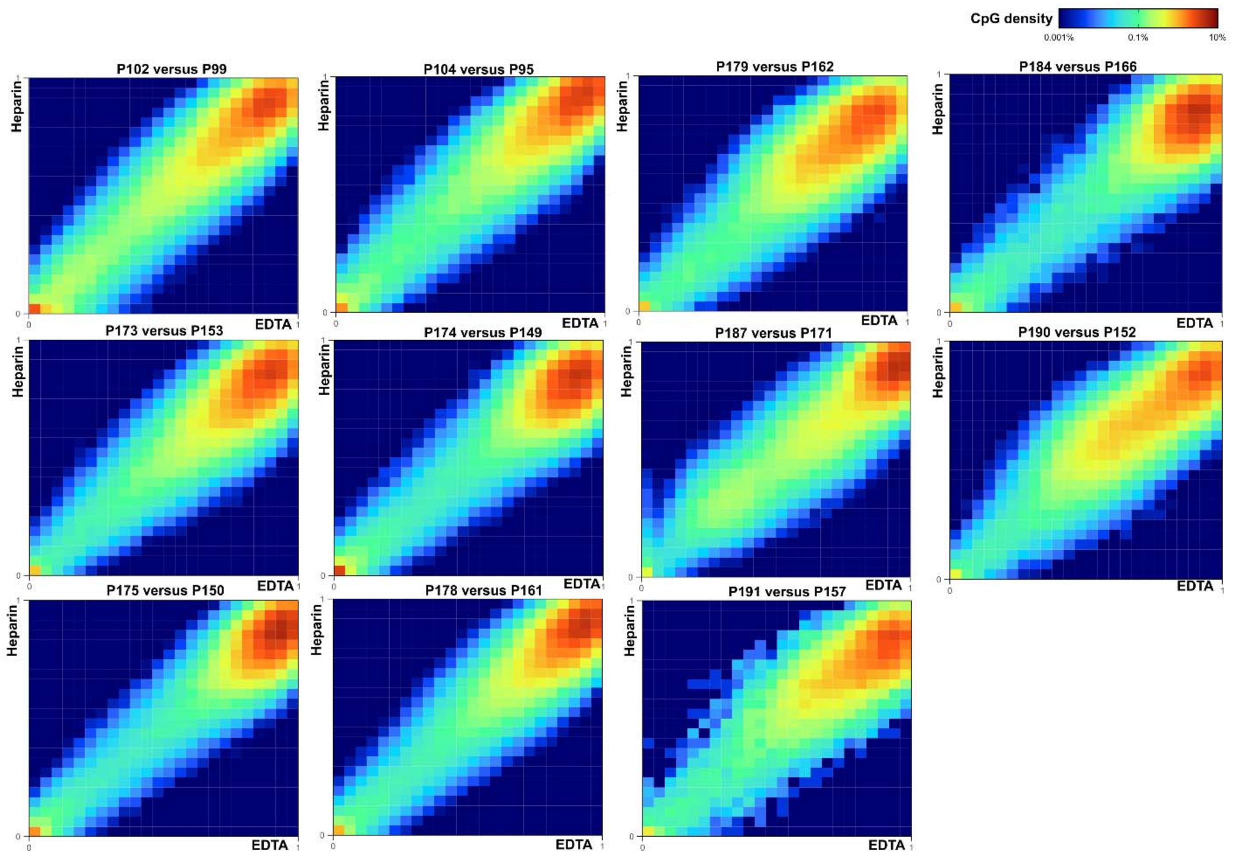

543

544 **Supplementary Figure 4. Methylation correlation across Hospital Cohort cases**

545 Heatmaps of methylation beta values in paired samples collected from 11 virus-positive patients

546 in the Hospital Cohort. One additional case is shown in Figure 5.

medRxiv preprint doi: https://doi.org/10.1101/2025.08.13.25333564; this version posted February 26, 2026. The copyright holder for this preprint (which was not certified by peer review) is the author/funder, who has granted medRxiv a license to display the preprint in perpetuity.
It is made available under a CC-BY 4.0 International license .

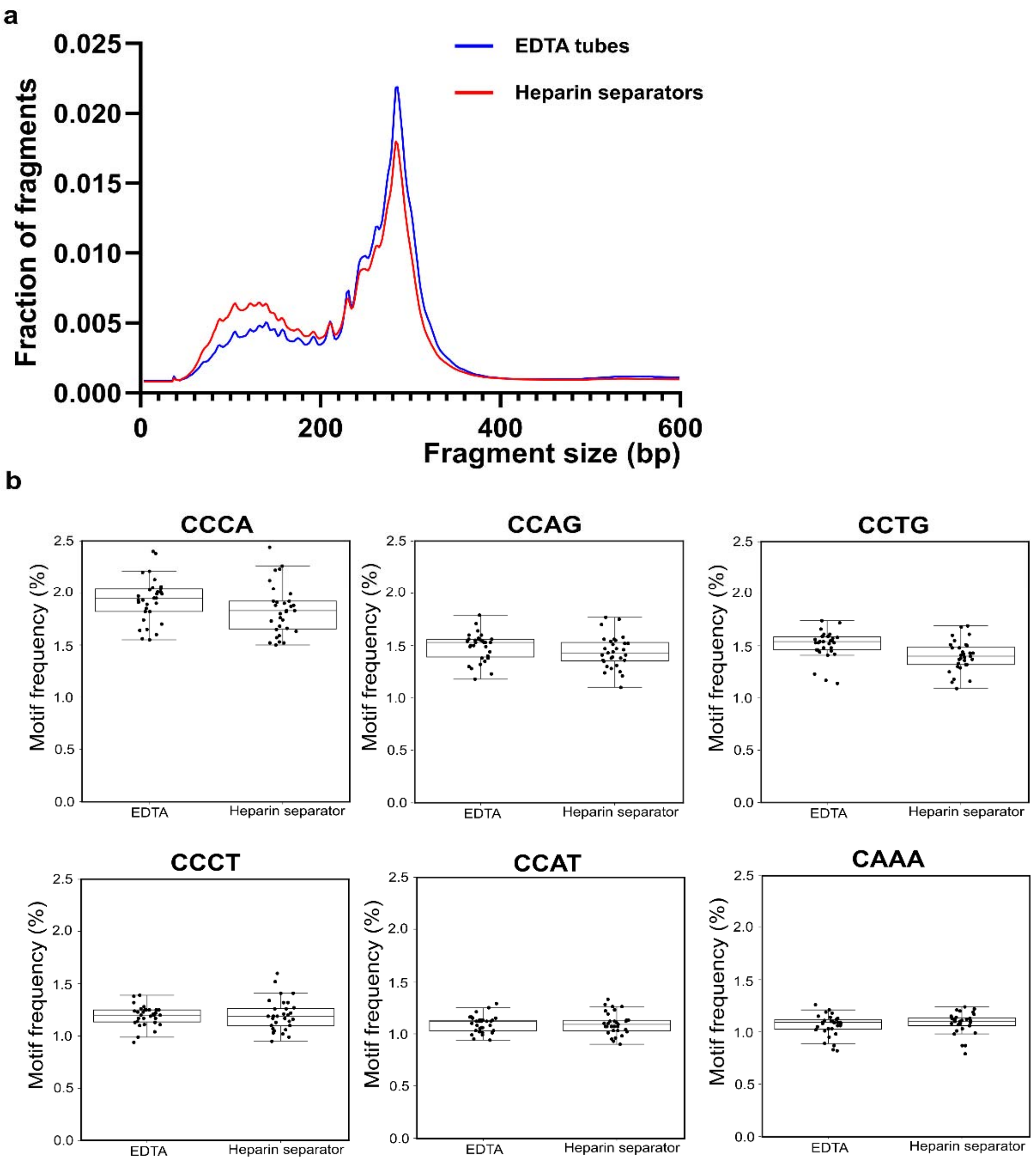

Supplementary Figure 5. Fragmentomic patterns of the Hospital Cohort

a. Average cfDNA fragment size distribution collected in EDTA tubes and heparin separators.

b. Average end motif frequency comparison between EDTA tubes and heparin separators, highlighting the top six motifs: CCCA, CCAG, CCTG, CCCT, CCAT, and CAAA.
